# Cys_2_/His_2_ Zinc-Finger Proteins in Transcriptional Regulation of Flower Development

**DOI:** 10.3390/ijms19092589

**Published:** 2018-08-31

**Authors:** Tianqi Lyu, Jiashu Cao

**Affiliations:** 1 Laboratory of Cell and Molecular Biology, Institute of Vegetable Science, Zhejiang University, Hangzhou 310058, China; 11716015@zju.edu.cn; 2 Key Laboratory of Horticultural Plant Growth, Development and Quality Improvement, Ministry of Agriculture, Hangzhou 310058, China; 3 Zhejiang Provincial Key Laboratory of Horticultural Plant Integrative Biology, Hangzhou 310058, China

**Keywords:** Cys_2_/His_2_ zinc-finger protein, flowering induction, floral organ morphogenesis, molecular mechanism, transcriptional regulation

## Abstract

Flower development is the core of higher-plant ontogenesis and is controlled by complex gene regulatory networks. Cys_2_/His_2_ zinc-finger proteins (C2H2-ZFPs) constitute one of the largest transcription factor families and are highly involved in transcriptional regulation of flowering induction, floral organ morphogenesis, and pollen and pistil maturation. Nevertheless, the molecular mechanism of C2H2-ZFPs has been gradually revealed only in recent years. During flowering induction, C2H2-ZFPs can modify the chromatin of *FLOWERING LOCUS C*, thereby providing additional insights into the quantification of transcriptional regulation caused by chromatin regulation. C2H2-ZFPs are involved in cell division and proliferation in floral organ development and are associated with hormonal regulation, thereby revealing how a flower is partitioned into four developmentally distinct whorls. The studies reviewed in this work integrate the information from the endogenous, hormonal, and environmental regulation of flower development. The structure of C2H2-ZFPs determines their function as transcriptional regulators. The findings indicate that C2H2-ZFPs play a crucial role in flower development. In this review, we summarize the current understanding of the structure, expression, and function of C2H2-ZFPs and discuss their molecular mechanism in flower development.

## 1. Introduction

Plants undergo two major postembryonic developmental transitions: infancy to adulthood, and adulthood to reproduction. Floral transition signifies the initiation of the reproductive growth stage of angiosperms. In *Arabidopsis*, flower development of dicotyledonous plants begins with flowering induction. Flowering induction is regulated mainly by four reaction pathways: photoperiod, vernalization, gibberellic acid, and autonomous pathways. In *Arabidopsis*, *FLOWERING LOCUS C* (*FLC*), which encodes a MADS-box transcription factor and acts as a critical gene for flowering induction, can inhibit the expression of flowering-time genes. In particular, *FLC* can inhibit the initiation of flowering during vernalization. Prior to vernalization, *FLC* inhibits flowering and prevents the shoot apical meristem (SAM) from transforming into a reproductive structure. After prolonged cold exposure, the expression of *FLC* is transcriptionally repressed, and plants can bloom. The activity of *FLC* is associated with chromatin-based gene regulation. FRIGIDA (FRI) and vernalization and autonomous pathways can modulate the local chromatin environment of *FLC*. FRI can directly activate the expression of *FLC*. However, the vernalization pathway can mask the activation of FRI on *FLC* [1]. The transformation from SAM into reproductive structures involves the interaction between a series of essential genes and *FLC*. Three flowering-time genes, namely, *FLOWERING LOCUS T* (*FT*), *SUPPRESSOR OF OVEREXPRESSION OF CO 1* (*SOC1*), and *FLOWERING LOCUS D*, can be regulated directly by *FLC* [2,3,4,5]. These molecules serve as effector genes of the floral organ for activating or inhibiting the expression of the downstream floral meristem (FM) and floral identity genes [6]. Therefore, *FLC* is a crucial factor that links flowering induction and floral organ morphogenesis. Most of the complex mechanisms involved have been reported. Nevertheless, the study of *FLC* can still be expanded considerably.

Flowers are individual reproductive structures produced by angiosperms and are the most complex plant structures. Floral organ formation starts after the induction. A typical dicotyledonous flower of *Arabidopsis* develops from four concentric circles. From outside to inside, the flower consists of four sepals, four petals, six stamens, and two fused carpels. The molecular mechanisms of each whorl of the floral organ have been illustrated using ABC and ABCE models [7,8]. FM emerges from the peripheral area of the SAM. The four whorls are all derived from the FM. FM will terminate its activity once it produces a certain number of floral organs; this process is regulated mainly by a homeodomain protein, WUSCHEL (WUS), and a floral homeotic protein, AGAMOUS (AG) [9]. Precise cell division and proliferation under the coordination of various transcriptional regulators are required to ensure that the flowers can be divided into four whorls of the visible floral organ and that plants can produce flowers that are similar in number and size. The molecular and genetic networks that establish the identity of floral organs have been extensively studied. However, for angiosperms, the precise transcriptional regulation of flower development remains unclear. After the formation of a floral organ with four divided whorls, the pistil and the stamen will develop further to maturity. The normal development of pollens and ovules can ensure the fertility of plants and complete double fertilization. At this stage, plants can accomplish the entire breeding process. 

Limited information is available on flower development by gene action, and the specific floral homeotic genes remain unidentified. Recent studies have focused on the genes that act upstream and downstream of these master regulators [10]. Flower development involves a complex transcriptional regulation. In addition, recent studies have demonstrated that transcriptional regulators containing MYB, zinc-finger (ZF), basic helix–loop–helix, MADS, and other DNA-binding domains perform a number of functions that were originally attributed to floral organ identity factors [11,12,13,14]. ZF is one of the important domains in the transcription factors of eukaryotic cells [15]; it is widely abundant in animals, plants, and microbes. ZF plays a key role in the regulation of gene repression or activation in yeast and the human body. In the human genome, nearly 1% of the sequences encode ZF-containing proteins, and nearly half of the transcription factors are Cys_2_/His_2_ ZF proteins (C2H2-ZFPs) [16]. Approximately 0.8% of the sequences encode C2H2-ZFPs in the *Arabidopsis* genome [17]. Therefore, C2H2-ZFPs constitute the largest group of DNA-binding domains in eukaryotic cells. C2H2-ZFPs can combine with DNA, RNA, and proteins and can therefore perform various biological functions. They participate not only in transcriptional regulation but also in chromatin regulation through unique locus modification and in RNA metabolism [18]. In 1992, Takatsuji cloned the first C2H2-ZFP gene of plants [19]; since then, the role of C2H2-ZFP in flower development has been extensively investigated. Gene expression analysis, transgene technology, and transcriptome sequencing have elucidated the precise role of C2H2-ZFPs in flower development. Studies in the past five years have gradually revealed the molecular mechanisms of C2H2-ZFPs involved in flower development. However, the existing data are spread across numerous works. Thus, a comprehensive review of the findings on C2H2-ZFPs in flower development will facilitate a better understanding of the formation process of plant flowers and a projection of potential future developments in this field.

## 2. Structure and Classification of C2H2-ZFPs during Flower Development

### 2.1. Specific and Diverse Structures of C2H2-ZFPs

C2H2-ZFPs are highly involved in transcriptional regulation and chromatin remodeling. C2H2-ZFPs rely mainly on their distinctive structures in identifying and integrating target sites. An independent C2H2-ZF domain contains a well-conserved DNA-binding structure and identifies specific target sites via its α helix [20]. The location of target genes by the C2H2-ZF domain is specific and diverse. In contrast to other structural classes of DNA-binding domains that typically offer a limited range of specificity, C2H2-ZF domains can specify a wide range of three-base-pair targets [21,22]. C2H2-ZF domains with completely different sequences can identify similar DNA-binding sites, and fingers with similar sequences can diverge in their preferences [20].

The majority of plant C2H2-ZFPs contain one or more ZF motifs. Meanwhile, other C2H2-ZFPs possess an invariant QALGGH motif in the ZF helices [23]. These proteins are collectively known as Q-type C2H2-ZFPs and constitute an independent subfamily [24]. The conserved QALGGH motif has not been reported in organisms other than plants, suggesting that this type of ZF protein may be involved in controlling unique plant life processes [15]. Mutations in the QALGGH sequence significantly affect the DNA-binding activity of ZF [23]. However, not all C2H2-ZFPs contain QALGGH domains. For example, the function and genome-wide targeting of RELATIVE OF EARLY FLOWERING 6 (REF6) require four C2H2-ZF domains, which directly recognize a CTCTGYTY motif [25]. Similar to REF6, EARLY FLOWERING6 (ELF6) and SUPPRESSOR OF FRIGIDA4 (SUF4) contain no QALGGH domains but still play significant roles in regulating floral transition [1,26,27,28]. In addition, REF6, ELF6, and Photoperiod sensitivity-14 (Se14) contain JmjN and JmjC domains, which cause these proteins to function as demethylases [29,30,31]. These properties indicate that domains other than the ZF motif also determine the function of C2H2-ZFP genes. Furthermore, researchers must still determine whether C2H2-ZFPs bind to target genes through motifs other than the QALGGH domain.

In plants, specific C2H2-ZFPs contain a highly conserved amino acid sequence (L/FDLNL/FxP) in their respective carboxy-terminal regions, and such a structure is designated as the ethylene-responsive element binding factor (ERF)-associated amphiphilic repression (EAR) motif [32]. The EAR motif is also available in other transcription factors, such as MYB, ERF, and NAC. Studies indicate that EAR domain is the smallest known repression domain and the first reported repression domain in plants. Whenever any individual residue within the EAR is replaced, the capability of the mutated peptide to repress transcription is reduced or eliminated, thereby producing a phenotype similar to the mutant [33]. These ZF proteins function as repressors, and their repression domains contain an EAR motif. In addition, the EAR domain can interact with the CTLH domain of TOPLESS (TPL)/TOPLESS-RELATED (TPR) [34]. As transcriptional regulators, TPL/TPR represses target gene expression by directly or indirectly combining with C2H2-ZFPs [35]. The EAR motif is the one of the most frequently observed, with 10% to 25% detection in transcription factors from diverse plant species. TLLLFR, R/KLFGV, and LxLxPP motifs are also transcriptional repressors. The TLLLFR motif is found solely in AtMYBL2. R/KLFGV and LxLxPP motifs account for less than 2% to 3% of the transcriptional repression [36]. At present, research on C2H2-ZFPs primarily focuses on the EAR motif. Future works should explore whether other types of repression motifs exist and participate in transcriptional repression of C2H2-ZFPs. In conclusion, C2H2-ZFPs are recruited by their unique ZF sequence and bound to the target gene locus. C2H2-ZFPs can also repress the expression of downstream genes via the EAR domain. Therefore, analyzing the structure of C2H2-ZFPs will elucidate their biological functions and the molecular regulatory mechanisms involved.

### 2.2. Classification of C2H2-ZFPs

Takatsuji et al. [19] cloned the first plant C2H2-ZFP gene *EFP1* in the study of *Petunia* petal development. In *Petunia*, more than 30 C2H2-ZFPs are found with the common QALGGH motif in the DNA recognition region and regarded as the EPF ZF family of this genus [23]. Computational analysis revealed that C2H2-ZFPs account for 2.3%, 3%, and 0.8% of the genomes in *Drosophila*, mammals, and yeast, respectively [37,38]. Genome analysis identified 176 and 189 C2H2-ZFP genes in *Arabidopsis* and the rice genome, respectively [17,39].

C2H2-ZFPs are generally classified according to four structures: tandem or dispersed [17,40], spacer regions [23], number of fingers [17], and QALGGH motif [41]. Such a classification approach is intuitive and straightforward. On this basis, the C2H2-ZFP family in *Arabidopsis* is further divided into three subgroups: A, B, and C. Particular focus is directed at the two largest and evolutionarily youngest subsets (A1 and C1), which are reportedly involved in transcriptional regulation. Subset A1 includes proteins with tandemly arranged ZF domains. By contrast, subset C1, which has been described as the EPF family in *Petunia*, consists of one isolated or two to five dispersed fingers and typically contains a QALGGH domain in the ZF helices. The subsets are further subdivided based on the different patterns of amino acids in the helices [17]. In the role of flower development of *Arabidopsis*, the C2H2-ZFPs feature diverse structures and belong to different subsets. For example, REF6 and ELF6, which are involved in flowering induction, are unique members of subset A2. CZS, SUF4, and LATE FLOWERING (LATE) also participate in flowering induction and belong to subsets A3, A4, and C1, respectively. SUPERMAN (SUP), KNUCKLES (KNU), RABBIT EARS (RBE), and JAGGED (JAG), which regulate floral organ development, are mainly distributed in subset C1. In rice, C2H2-ZFPs possess two main types of ZF domains (named C and Q). Q-type ZFs contain conserved QALGGH motifs, whereas C-type ZFs are found in other organisms [39].

The development of high-throughput sequencing technology and genome-wide analysis technology has enabled the analysis of the C2H2-ZFP family of several species. In plants, genome-wide analysis detected 124 C2H2-ZFPs in foxtail millet, 122 in sorghum [42], 109 in poplar [43], 211 in maize [44], 188 in tobacco [45], and 321 in soybean [46]. In foxtail millet, 78% of C2H2-ZFPs comprise QALGGH motifs and are designated as Q-type ZFs [42]. Soybean C2H2-ZFP genes are defined and classified into 11 distinguishable subsets on the basis of arrangements, numbers, and types of C2H2-ZF domains. Phylogenetic and gene ontology analyses further confirmed the rationality of classification [46]. Similar to *Arabidopsis*, the C2H2-ZFP family of maize is divided into three subgroups (A, B, and C) [44]. Englbrecht et al. [17] believed that single- and two-fingered proteins are evolutionarily older, and more than two-fingered proteins were derived. Among these proteins, the ancient subgroups are typically involved in RNA metabolism and chromatin remodeling. The younger subgroups are mainly involved in transcriptional regulation; thus, they receive particular research focus. The present knowledge is inadequate for categorizing C2H2-ZFPs by function. However, studying the structure and classification of C2H2-ZFPs in eukaryotes will offer insights into their capability to combine with DNA in transcriptional regulation.

## 3. Transcriptional Regulation of C2H2-ZFPs in Flowering Induction

**Figure 1:**
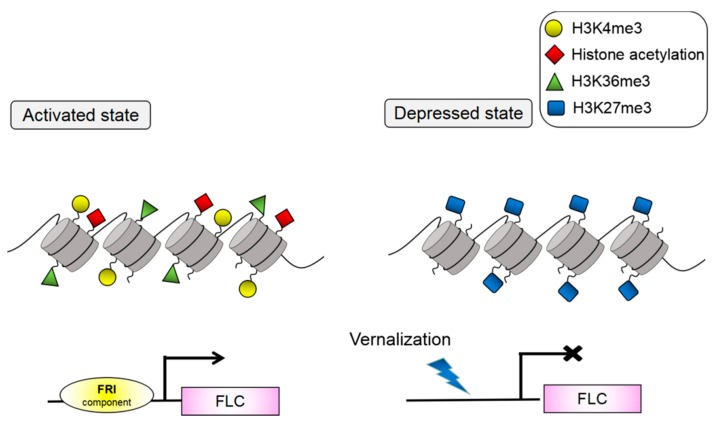
Histone modification of *FLC* locus. FRI can recruit other DNA-binding proteins and form complexes with one another. The complexes can combine with the cis-element of the *FLC* promoter, transforming *FLC* into a transcriptional activation state. At this time, *FLC* can present an active state marked by H3K36me3, H3K4me3, and histone acetylation. Vernalization can mask the regulation of FRI on *FLC*. *FLC* can present a silent state marked by H3K27me3.

**Figure 2:**
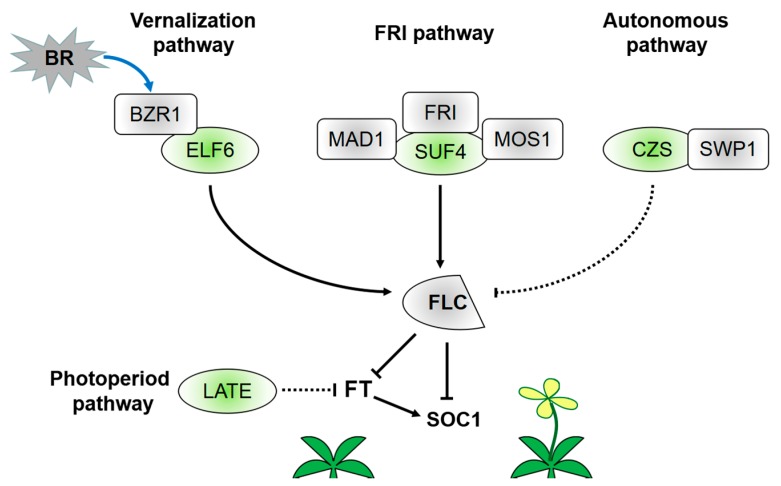
Regulatory network of C2H2-ZFPs in flowering induction. As demethylases or methyltransferases, C2H2-ZFPs are involved in histone modification of the *FLC* locus and flowering induction. C2H2-ZFPs can participate in vernalization, FRI regulation, and autonomous regulation pathways of *FLC*. They can also repress *FT* expression in the photoperiod pathway. ELF6 can perform the function of H3K27me3 demethylase and interact with BZR1. Then, the protein complex can activate *FLC* expression and inhibit flowering transition. SUF4 is required in the positive regulation of *FLC* by FRI. Both MAD1 and MOS1 can interact with SUF4. Therefore, SUF4 links the cell cycle and flowering induction. CZS interacts with SWP1. Subsequently, the protein complex participates in histone deacetylation and H3K9/H3K27 methylation. LATE may repress *FT* expression in the photoperiod regulation pathway.

C2H2-ZFPs’ expression in the SAM or leaves has been demonstrated to be involved in flowering induction. Loss of their function results in delayed or early flowering (Table S1). As demethylases or methyltransferases, C2H2-ZFPs participate in histone modification of *FLC* and are associated with flowering induction. Histone modification is a crucial function in chromatin regulation of *FLC* and coordinates the initiation and extension of transcription. That is, when H3K4me3/H3K36me3 and histone acetylation marks exist, *FLC* is activated, whereas *FLC* is silent when H3K27me3 marks exist [47] (Figure 1). These markers represent two opposing, mutually exclusive histone modification states. Such feedback mechanisms can provide a robust bistability and facilitate transcriptional regulation. C2H2-ZFPs can act as H3K27me3 demethylases to activate *FLC* transcription (Figure 2). REF6 is the first reported H3K27me3 demethylase in plants [48]. *REF6* is highly expressed in the SAM region and root tips but lowly expressed in cotyledons, leaves, and root axes, especially along vascular tissues. The lack of function of *REF6* will delay flowering in *Arabidopsis*. This expression pattern of *REF6* is the same as those of *FLC* and other genes affecting *FLC* expression [26,49,50]. REF6 can act as a *FLC* repressor; however, *FLC* is an indirect downstream target gene of REF6 [27]. ELF6 and REF6 are most homologous to each other in *Arabidopsis*. By contrast, the ELF6 expression pattern considerably differs from that of REF6 [26]. Studies showed that ELF6 can participate in the *FLC* activation pathway as an H3K27me3 demethylase [1]. Before vernalization, the H3K27me3 level is low, consistent with the enriched expression of ELF6 at the *FLC* locus. In warm environments, the H3K36 methyltransferase SET DOMAIN GROUP 8 (SDG8) is more enriched over the promoter of *FLC*. SDG8 and ELF6 influence each other’s localization at the *FLC* locus. This coupling facilitates the bistability of opposing histone states. ELF6 can directly interact with BRASSINAZOLE-RESISTANT 1 (BZR1) and be recruited at the *FLC* to activate the *FLC* expression [51]. The ZF domain of ELF6 and REF6 can interact with BRI1-EMS-SUPPRESSOR 1 (BES1)/BZR2 [52]. Furthermore, BZR1, rather than BES1, transduces brassinosteroid (BR) signals at the *FLC* locus and promotes *FLC* expression [51]. The latest findings reveal that BR signaling inhibits floral transition.

The activation effect of C2H2-ZFPs on the chromatin modification of *FLC* is connected with increasing H3K4me3 level at the *FLC* locus. SUF4 can induce the recruitment of H3K4me3 to activate *FLC* and is critical to the positive regulation of *FLC* by FRI (Figure 2) [28,53]. MODIFIER OF snc1 (MOS1) is a negative regulator of plant immunity with no DNA-binding capacity; however, it can promote flowering by directly interacting with SUF4 and inhibiting SUF4 to negatively regulate the *FLC* expression [28]. Mitotic arrest deficient 1 (MAD1) is an *Arabidopsis* spindle assembly checkpoint complex composed of a positive regulator of *FLC* in flowering induction and endopolyploidization via genetic interaction with MOS1. SUF4 can interact with MAD1 to regulate flowering time and endopolyploidization in the same pathway. That is, MOS1, MAD1, and SUF4 act as regulators of endopolyploidization and flowering induction. This study indicates that C2H2-ZFPs crucially link the plant cell cycle and timing mechanism of reproductive transformation. At present, limited works explore the regulation mechanism of *FLC* in the autonomous flowering pathway. Encoding a plant-specific C2H2 ZF-SET domain protein, CZS is the first histone methyltransferase reported to induce *FLC* silencing through an autonomous pathway [54]. The SET domain is found in all proteins that function as histone methyltransferases [55]. CZS interacts with SWIRM-domain polyamine oxidase protein 1 (SWP1) to facilitate the deacetylation of histones and methylation of H3K9/H3K27 after complex formation [54]. The co-repressor complex also exhibits a fine-tuning role in autonomous regulation of *FLC*.

Not all C2H2-ZFPs involved in flowering induction are associated with *FLC* locus modification. Among these C2H2-ZFPs, LATE participates in the photoperiod pathway of *FT* (Figure 2). LATE can interfere with the activity of *FT* upstream regulatory factors to block the photoperiod flowering response. LATE expression in leaf vasculature can inhibit the long-day response of *FT* [56]. However, studies must still determine whether LATE regulates *FT* by directly binding to the *FT* promoter or combining with other already known *FT* repressor complexes. Moreover, limited information is available on the role of LATE in this specific mechanism. In contrast to *Arabidopsis*, rice is a short-day model plant and therefore contains no *FLC* ortholog [57,58]. However, *Heading date 3a* in rice, similar to the *FT* ortholog in *Arabidopsis*, plays a major role in the photoperiod regulation pathway [59,60]. In rice, C2H2-ZFP genes *Suppressor of rid1*, *Rice Indeterminate1* (*RID1*), and *Se14* also participate in the photoperiod pathway by directly or indirectly acting on another florigen gene *RFT1* [31,61,62].

C2H2-ZFPs’ expression in SAM suggests that these proteins may be involved in the construction of floral organ models (Table S1). REF6 can act as H3K27me3 demethylase and regulate floral organ development and boundary formation [29]. The C2H2-ZF domain of REF6 can directly recognize a CTCTGYTY motif and is required for REF6 demethylase activity. REF6 directly binds and activates the expression of *CUP-SHAPED COTYLEDON 1* (*CUC1*), which is a key gene that regulates the cotyledon and other organ boundaries [29]. After binding to the target gene, REF6 promotes the recruitment of BRAHMA (BRM) (SWI/SNF-type chromatin-remodeling ATPase). BRM reinforces the function of REF6 as a H3K27 demethylase [25]. *LATE* is another inhibitor of floral organs. Ectopic *LATE* expression in the SAM can impair the transformation of this tissue from the vegetative to reproductive phase and interfere with the upregulation and sustained expression of the FM identity gene *SOC1* [56]. *RID1* is also expressed in the SAM and acts as a master switch for the phase transition from vegetative to reproductive growth in rice; *RID1* mutation will inhibit flowering [61,63]. The available data on LATE and other C2H2-ZFPs provide inconsistent detailed information on its functions. Further investigations must be conducted on genetic functions and transcriptional regulation of C2H2-ZFPs. Overall, these findings indicate that C2H2-ZPFs are versatilely and widely involved in numerous processes of flower development. Therefore, flowering induction and floral organ formation are possibly related in a complex regulatory network.

## 4. Transcriptional Regulation of C2H2-ZFPs in Floral Organ Development

The over-ground part of all plants originates from the proliferation and differentiation of the SAM. After flowering induction, the periphery of the SAM develops into FMs. In turn, FMs generate four developmentally distinct whorls that constitute the discrete floral organs. The identity of each floral organ is determined by specific floral homeotic genes; the molecular network that identifies floral organ recognition has been extensively studied [64]. Nevertheless, the ABCE model is still inadequate for comprehending floral organ formation. Recent studies have revealed that the upstream or downstream regulatory factors of these major floral homeotic genes also perform the same function and participate in floral organ development [10]. However, limited information is known about the mechanisms by which the flower is divided into four developmentally distinct whorls, particularly the mechanism by which boundaries are established between different floral organs. C2H2-ZFPs are bound by complex interactions among transcription factors, epigenetic modification, cell proliferation, and hormonal regulation. Studies have traditionally examined the effect of C2H2-ZFPs on the establishment of floral organ models. Research on the role of C2H2-ZFPs in cell proliferation and differentiation clarify this issue satisfactorily.

**Figure 3:**
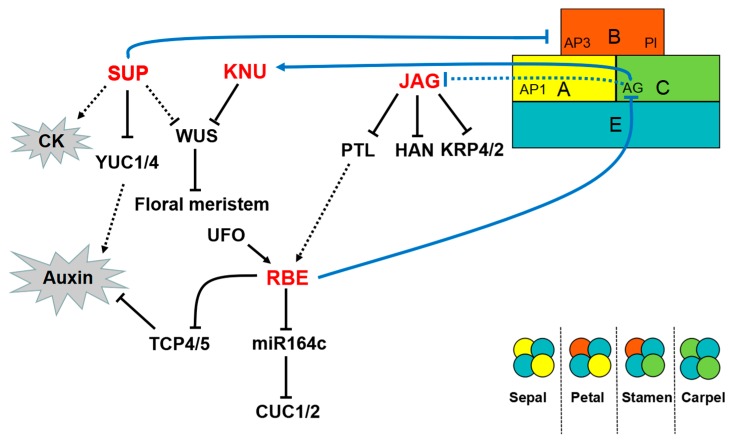
Regulatory network of C2H2-ZFPs in floral organ development. In the ABCE model of flower development, A class genes specify sepals in whorl 1; A and B class genes specify petals in whorl 2; B and C class genes specify stamens in whorl 3; C class genes define carpels in whorl 4. E class genes are active in all four whorls. *SUP* and *KNU* directly or indirectly repress WUS and terminate the FM activity. *SUP* can repress the expression of B function genes, *AP3* and *PI*. *SUP* can repress YUC1/2 and participate in floral organ development by affecting auxin signaling, whereas SUP may regulate CK signaling. *KNU* is directly activated by the C class gene *AG*. *AG* may repress *JAG*, whereas JAG further inhibits *PTL*, *HAN*, *KRP2*, and *KRP4*. Thus, JAG is a direct regulator of the cell cycle during floral organ tissue growth. RBE is involved in the MIR164–CUC regulation pathway. *UFO* acts on the upstream of *RBE*. RBE can also repress *TCP4* and *TCP5*.

C2H2-ZFPs affect flower development by acting on the primordium of floral organs (Table S2). The *SUP* gene encodes a transcriptional repressor with a C2H2-ZF DNA-binding domain and an EAR repression domain [33,65,66,67]. In *Arabidopsis*, SUP controls the stamen primordia, carpel primordia, and ovules during flower development [33,67,68,69]. SUP is specifically required to balance the proliferation and create a boundary between the whorl 3 and 4. The boundary-specific function of SUP is to repress the expression of B function regulators, namely, *APETALA 3* and *PISTILLATA*, by modulating auxin- and cytokinin (CK)-regulated processes (Figure 3) [13,67,70]. Several *SUP* homologs have been identified. *SUP* performs conserved functions in tobacco, *Silene latifolia*, and rice at different expression levels (Table S2) [71,72,73].

In *Arabidopsis*, the activity of FM cells is maintained only at the initial stages of flower development and is subsequently terminated by *WUS* at the appropriate time. In addition to regulating organ boundaries, *SUP* also regulates the FM activity. *SUP* may indirectly regulate *WUS* with independence of the C class gene *AG* [13]. *SUP*, *AG*, and *CLAVATA3* coordinate and monitor the FM activity in partially redundant pathways [74]. The FM-activity-regulating function of SUP is related to auxin-regulated processes. SUP can act as an active repressor of auxin biosynthesis and recruit the Polycomb group (PcG) component CURLY LEAF to directly repress the auxin biosynthesis genes *YUCCA1* and *4* (*YUC1/4*) (Figure 3) [75]. In *sup* mutants, ectopic auxin activity delays the termination activity and increases the size of FM [75]. The KNU transcription factor has also been shown to regulate the FM activity. *KNU* encodes a SUP-like protein and contains an EAR-like active repression domain [76]. In contrast to *SUP*, *KNU* must be assisted by *AG* to regulate WUS (Figure 3). AG directly binds to the *KNU* promoter but induces *KNU* with a time delay regulated by epigenetic modification. This induction process requires approximately two days and is associated with the timing mechanism of releasing histone methylation inhibition at the *KNU* locus. Prior to stage 3, the *KNU* locus is covered by H3K27me3, which is maintained by PcG. H3K27me3 is produced by PcG during cell division. Upon directly binding to the *KNU* promoter, AG competes with and expels PcG from the *KNU* locus. Consequently, the inhibitory state of *KNU* is relieved. The cells divide and inhibit *WUS* expression, and the FM activity is terminated at the appropriate time [77]. Such a timing mechanism guided by *KNU* is fundamental in balancing cell proliferation and differentiation during flower development. This mechanism reveals the relationship between transcriptional regulation and epigenetic control in plant stem cell proliferation and suggests that cell division plays a vital role in the morphological changes during floral organ development.

*RBE*, which encodes a C2H2 ZF protein that is closely related to *SUP*, causes the formation of petal primordium [78]. RBE may act downstream of an A function gene, *APETALA1* (*AP1*), and downstream of *PETAL LOSS* (*PTL*), as *RBE* is unexpressed in *ap1-1* and *ptl-1* [78]. In addition, RBE can maintain the boundary between the whorl of 2 and 3 and inhibit the expression of the C class gene *AG* only within the second whorl [79]. The gene *UNUSUAL FLORAL ORGANS* (*UFO*), which degrades the repressor of cell proliferation, is the upstream regulator and essential for maintaining regular *RBE* expression. In addition, UFO promotes the initiation and development of petal primordia by repressing *AG* expression. UFO and RBE act on the same pathway to regulate the development of whorl 2 [79,80]. In *Arabidopsis*, three microRNA164 genes (*MIR164a*, *b*, and *c*) regulate the expression of CUC1 and CUC2, which are the primary regulators involved in floral organ boundary specification [81,82,83]. RBE can regulate the expression of these microRNA164 genes. RBE also directly binds to the promoter and negatively regulates *MIR164c* expression. That is, RBE participates in the MIR164–CUC regulation pathway and influences floral organ development (Figure 3) [84]. At the early stage of petal development, *RBE* can directly and negatively regulate the growth inhibitor genes *TEOSINTE BRANCHED1/CYCLOIDEA/PCF 4* (*TCP4*) and *TCP5* to promote cell proliferation and growth of the petal primordia. Although both *TCP4* and *TCP5* inhibit cell proliferation, *TCP4*, rather than *TCP5*, is the target gene of miR319. RBE and microRNA319 regulate *TCP4* at the transcriptional and post-transcriptional levels, respectively. In turn, *TCP4* regulates the size and shape of *Arabidopsis* petals [85,86]. Furthermore, PTL and TCP4 are crucial in auxin regulation [87,88,89]. The transcriptional regulation of RBE in whorl 2 is associated with hormonal regulation.

Similar to RBE, JAG causes the formation of the petal primordium. JAG, together with its paralog NUBBIN (NUB), controls the growth of leaf margins and floral organs, particularly in their distal regions [90,91]. NUB acts redundantly with JAG to promote the differentiation of carpels and stamens. Researchers propose that JAG can stimulate organ growth by promoting cell proliferation. *JAG* induces complex cellular behavior, which includes cell proliferation and elongation, changes in cell size homeostasis, and regulation of anisotropic growth [91,92]. In the mutant *jag*, the entire distal part of the petal can be eliminated, resulting in a jagged edge. The edges of *jag* sepals and leaves are also serrated [91]. Petal development involves a divergent polarity field with growth rates. Therefore, *JAG* may regulate the growth rate of petals through auxin regulation [93]. *JAG* can directly inhibit the expression of *PTL*, which encodes a transcription factor that acts on the upstream of auxin signals that initiate petal development. In addition, *JAG* is a direct target gene of the C class gene *AG* [94]. Cell cycle progression can promote plant organ growth. Schiessl et al. [92] confirmed that JAG can directly mediate the cell cycle. Their results showed that JAG directly repressed cell-cycle-dependent kinase genes *KIP RELATED PROTEIN 4* (*KRP4*) and *KRP2*, which control the transition to the DNA synthesis phase of the cell cycle (Figure 3). Mutations *krp2* and *krp4* suppressed the *jag* defects during petal growth. These findings are consistent with the role of JAG in regulating cell proliferation. JAG also influences the coordination between cell growth and cell cycle during floral organ development by directly inhibiting *BREVIPEDICELLUS* (*BP*) and *BELL 1*, which are regulators of the meristem [95]. *HANABA TARANU* (*HAN*), which is a key regulator of the FM and organ primordia boundaries, can interact with or is directly inhibited by JAG. However, Ding et al. [96] subsequently observed that *JAG* can be directly activated and interact with HAN. Thus, new experimental methods and protocols must be developed to analyze the role of JAG in floral organ growth. The regulation of JAG serves as a starting point to address the molecular mechanisms underlying other key aspects of floral organ morphogenesis.

C2H2-ZFPs are less involved in regulating floral organ maturation than in regulating flowering induction and floral organ development. For example, the expression patterns of C2H2-ZFP genes, such as *BcMF20*, *ZPT2-10*, and *ZPT3-3*, indicate their role in the development of pollens and pistils (Table S3). However, the related research remains at its infancy. The role of *TAPETUM DEVELOPMENT ZINC FINGER PROTEIN1* (*TAZ1*) in microspores and tapetum, given its significant involvement in pollen development, has been extensively studied. *TAZ1* may be a component of *MALE STERILITY 1* (*MS1*) that possibly participates in the development of tapetum and regulates the fertility of pollens [97]. Tapetum development and degeneration are strictly relevant to microspore development. To date, in-depth studies on tapetum have been conducted. Aside from *MS1*, a number of genes are also involved in the programmed cell death of the tapetum. Nevertheless, further investigations are still needed to elucidate the specific regulation of C2H2-ZFPs in tapetum development. *MEiosis-associated Zinc-finger protein 1* (*MEZ1*) acts on the meiotic process. Silencing of *MEZ1* leads to aberrant meiosis and pollen abortion in *Petunia* [98]. *DUO POLLEN 1 (DUO1)-ACTIVATED ZINC FINGER 1*/*2* are two target genes of the germline-specific protein DUO1, and they participate in the germ cell division regulated by DUO1. These target genes promote germ cell division by regulating the accumulation of mitotic cyclin CYCB1;1, which promotes the G2-to-M phase transition [35]. The involvement of other mitotic cyclins in germ cell division remains unclear. At present, several genes, including *NTT*, have been detected in the development of the replum. *NTT* negatively regulates the expression of *FRUITFULL* and participates in dehiscence of fruit to reduce seed loss and increase yield [99]. NTT can bind to the *BP* promoter and activate *BP* expression in the replum. Furthermore, *NTT* can interact with itself and REPLUMLESS to participate in fruit development [100]. All these studies suggest that C2H2-ZFPs possibly play a more significant role in floral organ maturation. These studies have addressed the relation between C2H2-ZFPs and floral organ maturation, and future works will clarify this interrelationship at the transcriptional regulation level.

## 5. Conclusions and Perspectives

The results of the discussed studies not only show that C2H2-ZFPs are highly involved in flowering induction, floral organ morphogenesis, and maturation, but also consolidate the available information on flower development process. C2H2-ZFPs participate in both histone modification of the *FLC* locus and the photoperiod regulation pathway of *FT*, thereby influencing flowering induction. C2H2-ZFPs can directly or indirectly regulate the ABC model function genes in floral organ development by affecting hormonal signaling in cell proliferation and division processes. Thus, we can better understand the formation of floral organs with four whorls. C2H2-ZFPs are also involved in pollen and pistil development. Despite the recent progress that has been attained as regards the effect of C2H2-ZFPs on flower development, major issues remain unclear. Several of these issues have been mentioned above, particularly how C2H2-ZFPs regulate the maturity of floral organs. For instance, although *TAZ1* and *BcMF20* have been proven to participate in pollen development, limited information is available on the transcriptional regulation between them and other already known factors. Thus, we intend to study the transcriptional regulation of C2H2-ZFPs in pollen development and aim to achieve good results soon.

In addition to C2H2-ZFPs, numerous transcriptional factors are involved in flower development. However, existing works have fully confirmed the unique and exclusive role of C2H2-ZFPs in this field. Regardless of whether C2H2-ZFPs participate in the fine regulation of flower development, ensuring that C2H2-ZFPs play a vital role in floral organ morphogenesis is a significant achievement. Thus, future works should explore the mechanism by which C2H2-ZFP participates in flower development by interacting with other factors. Relevant studies have been limited to only a few model plants, particularly *Arabidopsis* and rice. The development of sequencing technology has enabled the analysis of C2H2-ZFP families in other species. Studies on C2H2-ZFPs in plants other than *Arabidopsis* will provide insights into the formation of general flowers. Studying the transcriptional regulation of C2H2-ZFPs in flower development of crops other than rice will contribute to crop breeding and development of agriculture. This work not only provides the necessary data for studying the function and regulation of C2H2-ZFPs in flower development but also ensures better comprehension of the construction of floral organs of various angiosperms. Although significant progress has been achieved in the study of C2H2-ZFPs in flower development, the critical issues about their function and expression remain unclear, particularly the involvement of C2H2-ZFPs in transcriptional regulation of flower development. Considerable progress is expected in this field in the future.

## Supplementary Materials

Supplementary materials can be found at http://www.mdpi.com/1422-0067/19/9/2589/s1.
