# Supplementary material for: Cys2/His2 Zinc-Finger Proteins in Transcriptional Regulation of Flower Development
Source: Int J Mol Sci. 2018 Aug 31;19(9):2589. doi: 10.3390/ijms19092589 (PMC6164605; doi:10.3390/ijms19092589)
Supplement: Supplementary file 1 [file ijms-19-02589-s001.pdf]

Table S1. C2H2-ZFPs expressed in shoot apical meristem or leaves and their functions.

Gene	Species	Expression	Gene function	Reference(s)
REF6	*Arabidopsis thaliana*	SAM and root tips	Flowering development	Noh et al., 2004
ELF6	*Arabidopsis thaliana*	Cotyledons and leaves	Flowering development	Noh et al., 2004
ID1	*Zea maize*	Leaves	Flowering development	Kozaki et al., 2004
SUF4	*Arabidopsis thaliana*	Leaves and inflorescence	Flowering development	Kim et al., 2006
CZS	*Arabidopsis thaliana*	Undefined	Flowering development	Krichevsky et al., 2007
Ehd2	*Oryza sativa*	Leaves	Flowering development	Matsubara et al., 2008
RID1	*Oryza sativa*	Root, mature leaves and SAM	Flowering development	Wu et al., 2008
LATE	*Arabidopsis thaliana*	Leaf vasculature, SAM	Flowering development	Weingartner et al., 2011
Ghd10	*Oryza sativa*	Leaves and panicle structures	Flowering development	Shikai et al., 2013
Se14	*Oryza sativa*	Undefined	Flowering development	Yokoo et al., 2014
SID1	*Oryza sativa*	Leaves	Flowering development	Deng et al., 2017

Table S2. C2H2-ZFPs expressed in floral meristem and their functions.

Gene	Species	Expression	Gene function	Reference(s)
SUP	*Arabidopsis thaliana*	Stamen and carpel primordia	Define the boundary between the stamens and carpels	Sakai et al., 1995; Prunet et al., 2017 Xu et al., 2018
NtSUP	*Nicotiana tabacum*	Undefined	Cell proliferation, expansion, and differentiation	Bereterbide et al., 2001
PhSUP1	*Petunia*	Anthers, funiculi, and organ primordia	Anther, ovule development	Nakagawa et al., 2004
SlSUP	*Silene latifolia*	Female flower	Female flower development	Kazama et al., 2009
CsSUP	*Cucumber*	Female flower buds and ovules	Stamen and fruit development	Zhao et al., 2014
RBE	*Arabidopsis thaliana*	Petal primordia	Petal development	Takeda et al., 2004
KNU	*Arabidopsis thaliana*	Carpel primordia, stamens, and ovules	Carpel development	Payne et al., 2004
JAG	*Arabidopsis thaliana*	Petal and organ primordia	Petal development	Schiessl et al., 2004;
SE	*Arabidopsis thaliana*	Meristems and organ primordia	Shoot development	Grigg et al., 2005
RA1	*Zea maize*	Second-order meristems	Inflorescence development	Vollbrecht et al., 2005
NUB	*Arabidopsis thaliana*	Leaf, stamen and carpel primordia	Stamen and carpel development	Dinneny et al., 2006
Sl1	*Oryza sativa*	Lodicules, stamens and carpel	Floral development	Xiao et al., 2009
OsJAG	*Oryza sativa*	Roots, leaves, stem, and all floral organs	Floral development	Duan et al., 2010
SlKNU	*Solanum lycopersicum*	Carpel primordia, stamens, ovules	Carpel development	Bollier et al., 2018

Table S3. C2H2-ZFPs expressed in pollen or pistil and their functions.

Gene	Species	Expression	Gene function	Reference(s)
ZPT2-10	*Petunia*	Pistil transmission tissue	Undefined	Kubo et al., 2000
ZPT3-3	*Petunia*	Pistil transmission tissue	Undefined	Kubo et al., 2000
TAZ1	*Petunia*	Pollen and tapetum	Degeneration of tapetum	Kapoor et al., 2002
MEZ1	*Petunia*	Pollen	Meiosis	Kapoor and Takatsuji, 2006
NTT	*Arabidopsis thaliana*	Carpel and replum	Transmitting-tract and replum development	Crawford et al., 2007
BcMF20	*Brassica campestris*	Pollen and tapetum	Pollen development	Han et al., 2011
DAZ1	*Arabidopsis thaliana*	Germ cell nuclear and cytoplasm	Germ cell division	Borg et al., 2014
DAZ2	*Arabidopsis thaliana*	Germ cell nuclear	Germ cell division	Borg et al., 2014
